# Eggshell calcified hydrocele sac: a case report

**DOI:** 10.1186/s13256-023-04076-9

**Published:** 2023-08-15

**Authors:** Yohannis Derbew Molla, Desyibelew Chanie Mekonnen, Cheru Lilay Gebrehiwot, Amanuel Kassa Tadesse, Samuel Addisu Abera, Lidetu Temeche

**Affiliations:** 1 Department of Surgery, College of Medicine and Health Sciences, University of Gondar, Gondar, Ethiopia; 2 Department of Pathology, College of Medicine and Health Sciences, University of Gondar, Gondar, Ethiopia

**Keywords:** Hydrocele, Calcification, Sac, Chronic irritation, Case report

## Abstract

**Introduction:**

Hydrocele, an abnormal fluid collection between parietal and visceral layers of the tunica vaginalis, is the commonest cause of scrotal swelling, and it affects all age groups. Calcification of hydrocele sac/wall is a rare clinical entity. The etiology of calcification of hydrocele sac is not clear, but most literature proposes that calcification is secondary to chronic irritation. Trauma and infectious diseases including *Schistosoma haematobium*, filariasis, and tuberculosis can also cause calcification of the hydrocele sac.

**Case presentation:**

A 74-year-old Ethiopian male patient presented with left side scrotal swelling of 3 years duration, which was initially small but progressively increased. He had no history of trauma, and he had no history of swelling on the contralateral side. Scrotal ultrasound (US) showed a large echodebrinous left-side scrotal collection with calcifications, bilateral testis appear normal in size, echogenicity, and color Doppler flow with the index of likely chronic hematocele. Therefore, with a diagnosis of left-sided calcified hydrocele, the patient was operated on and the calcified sac was excised and sent for histopathology. Finally, the patient was discharged improved after 2 days of hospital stay.

**Conclusion:**

Calcification of the tunica vaginalis is very rare and is probably due to chronic irritation of the wall from the coexisting hydrocele. Surgical excision of calcified hydrocele sac is the treatment of choice.

## Introduction

Hydrocele, an abnormal fluid collection between parietal and visceral layers of the tunica vaginalis, is the commonest cause of scrotal swelling, and it affects all age groups. Hydrocele may be idiopathic (primary) without predisposing lesion (risk factors), or secondary due to inflammation of the epididymis, epididymo-orchitis, tumors, trauma, surgical operation, torsion, or infarction. Abnormal collection of serous fluid in between two layers of tunica vaginalis in hydrocele can be either due to excessive secretion or because of poor absorption of fluid [1]. Calcification of the hydrocele sac is a rare complication. In 1935, Kickham published a case of calcified hydrocele of the tunica vaginalis testis, and that was the first reported case of a calcified hydrocele sac. Eggshell calcification of the scrotum may indicate a state of chronic infection within the hydrocele sac, like in patients with filariasis [2]. Although the exact etiology of calcification of the hydrocele sac is not known, it could be secondary to chronic irritation, trauma, and infectious diseases including *Schistosoma haematobium* [1, 3]. Here we present a case of eggshell calcification in a 74-year-old male patient.

## Case presentation

A 74-year-old Ethiopian male patient presented with left-side scrotal swelling of 3 years duration, which was initially small but progressively increased to attain the current size which makes his daily activities cumbersome. He had no history of trauma, and he had no history of swelling on the contralateral side or penis. He had no swelling in the groin or any other site. He had no history of diabetes, hypertension, asthma, or any known drug allergy. He had no history of previous surgery. He had a history of river water contact but no history of bloody diarrhea or bloody urine. He had no history of chronic cough, contact with a patient with chronic cough, or known pulmonary tuberculosis patient. He is a farmer and had no history of occupational exposure to chemicals. On examination, his vital signs were within normal limits. He had a clear and resonant chest. There was a huge, 17 × 12 cm firm to hard left-side scrotal swelling all over the left scrotum, and the trans-illumination test was negative. The right side was unremarkable. Otherwise, the rest of the examinations were unremarkable.

**Fig. 1:**
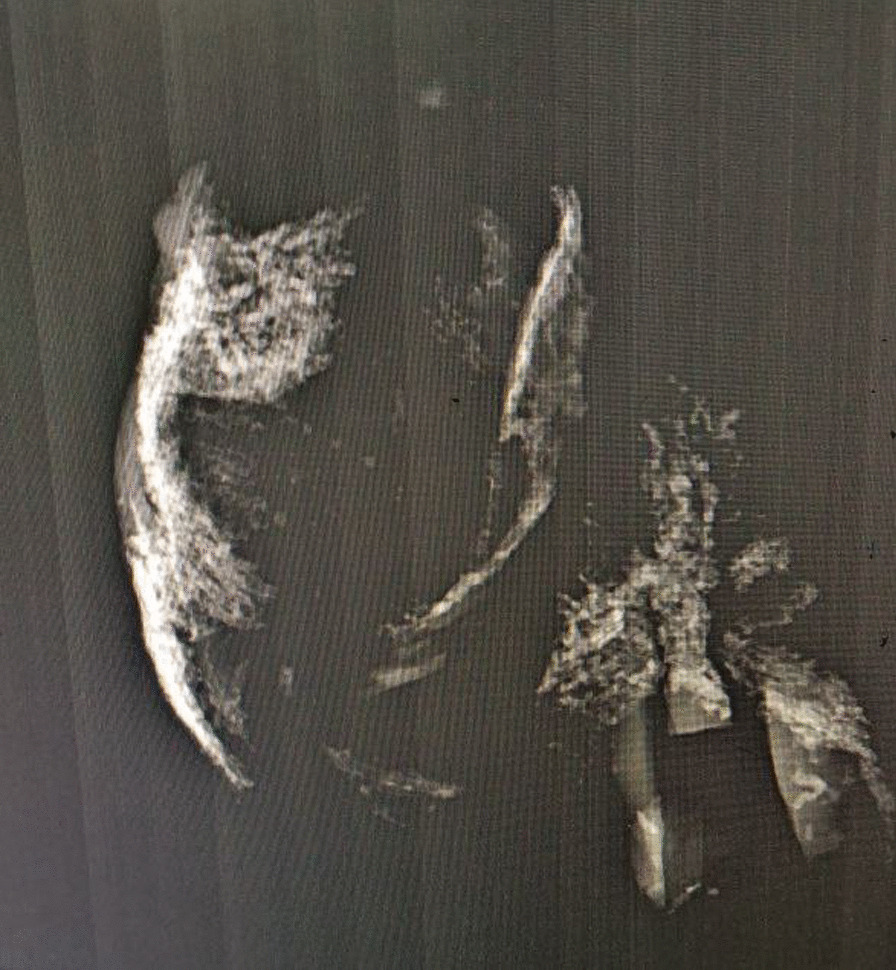
X-ray of the excised hydrocele sac showing extensive calcification of the wall

**Fig. 2:**
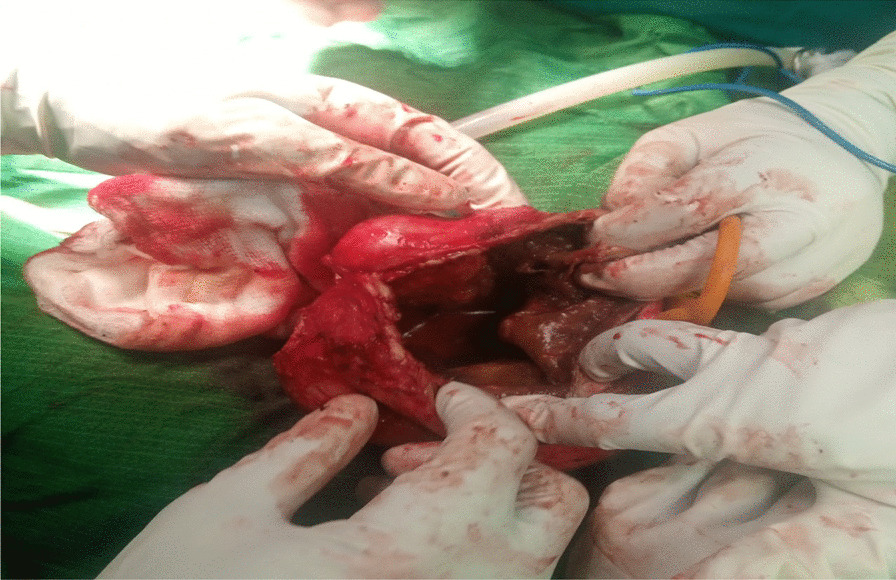
Intraoperative picture of the calcified tunica vaginalis

**Fig. 3:**
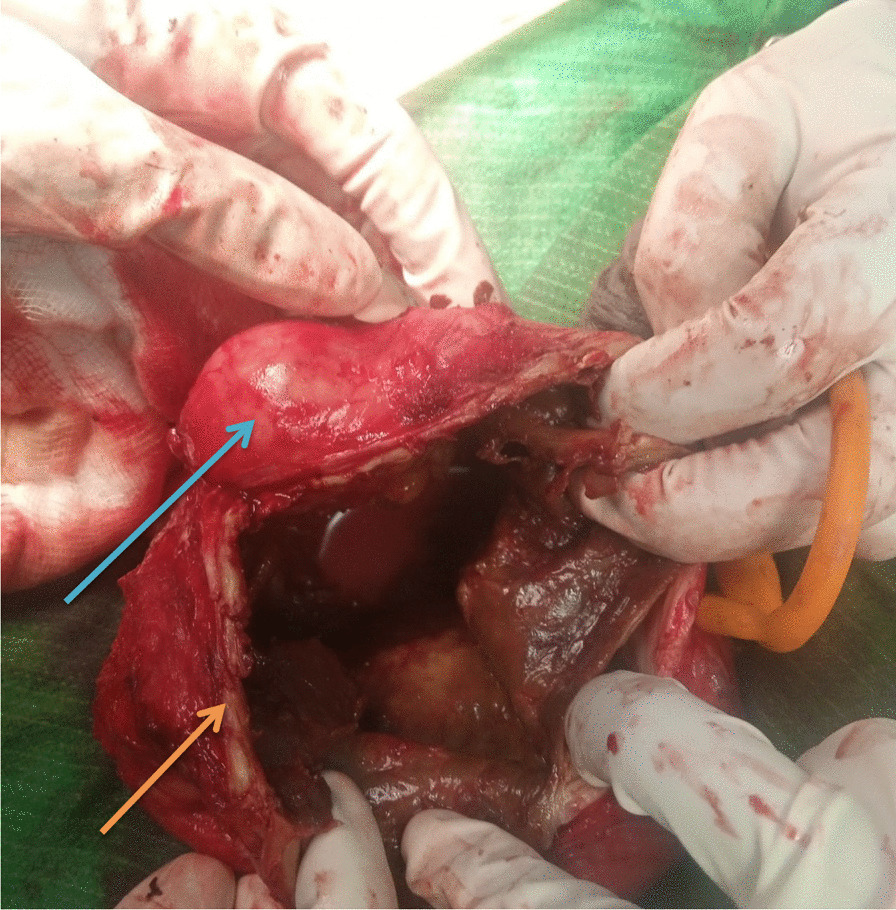
Another intraoperative picture; the blue arrow shows the testis, and the pink arrow shows calcified sac wall

**Fig. 4:**
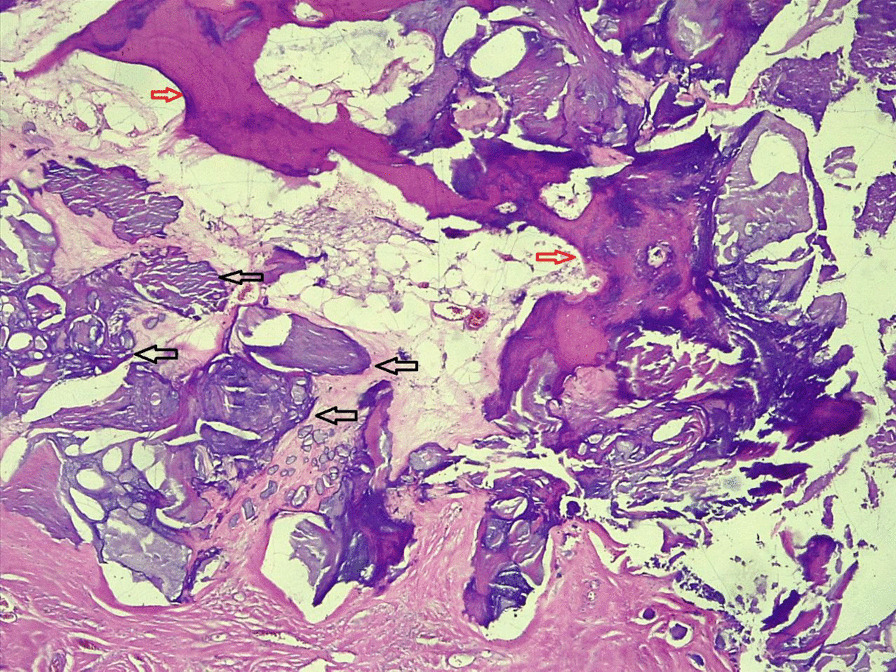
Histopathology section (40× magnification) showing a cyst wall without cyst lining showing fibrosis and extensive calcification (black arrow shows calcification; red arrow shows ossification)

**Fig. 5:**
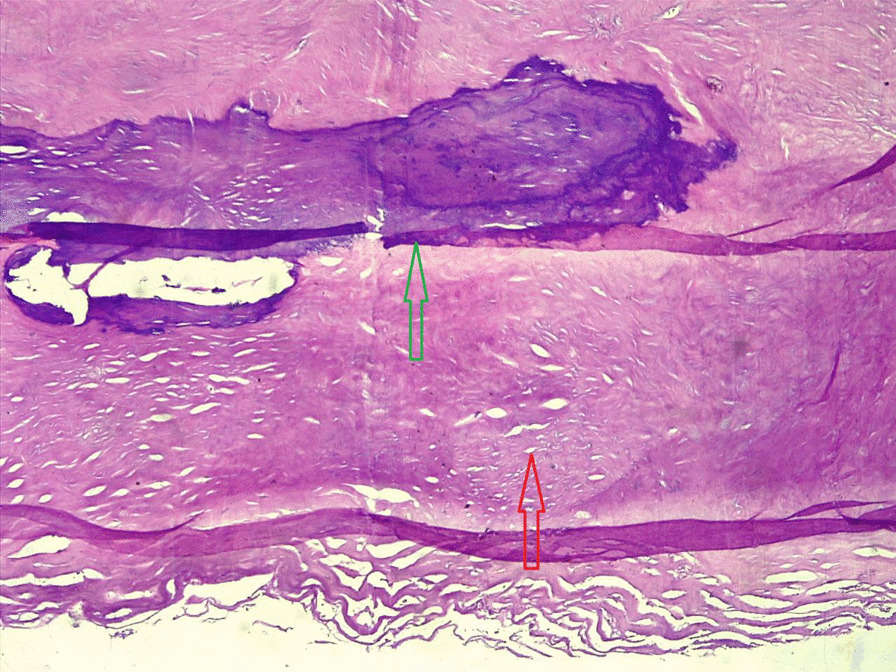
Histopathology section showing eggshell calcification (green arrow shows calcification and red arrow shows thickened fibrotic wall)

**Fig. 6:**
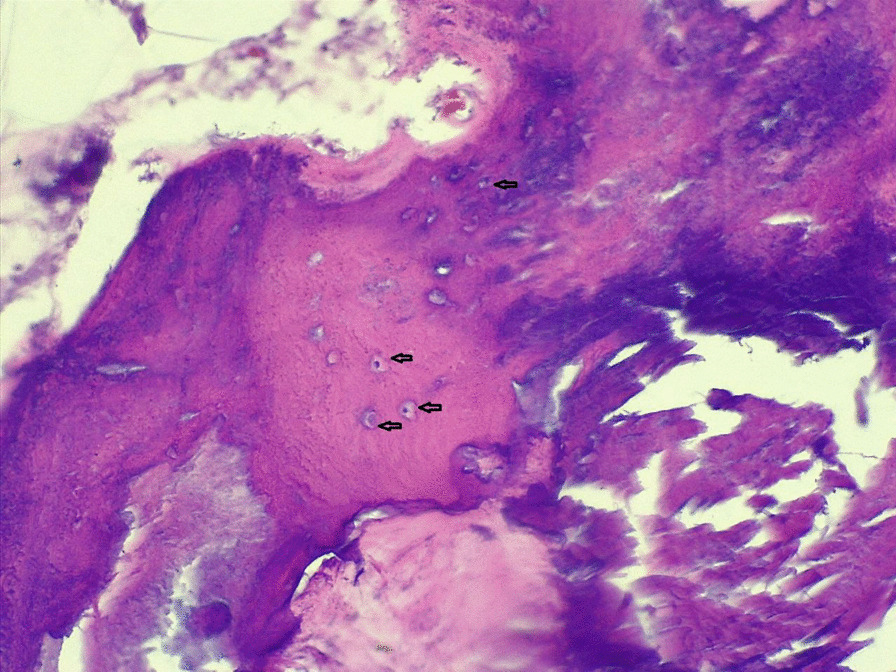
Histopathology (magnification 400×) showing osteocytes in three lacunae (arrows)

On investigation, his complete blood count was normal with no eosinophilia, urinalysis showed no chyluria, proteinuria, or hematuria, and organ function tests were within normal limits. Scrotal ultrasound (US) showed a large echodebrinous left-side scrotal collection measuring 15 × 10 cm having internal dispersed calcifications; bilateral testes appear normal in size, echogenicity, and color Doppler flow, no lymphatic obstruction, or filarial dance sign with the index of likely chronic hematocele. We did not order preoperative X-ray as it would not change our management, but we took an X-ray of the excised tissue and it showed eggshell-like calcified wall (Fig. 1). Therefore, with a diagnosis of chronic hematocele with calcification, the patient was kept NPO for 8 hours and operated on under spinal anesthesia and supine position by a urologist and general surgery residents. Intraoperatively, there was 800 ml of non-foul-smelling, turbid fluid within well-formed and calcified tunica vaginalis (Figs. 2, 3). There was gritty sound upon opening the tunica vaginalis. Therefore, a left-side hydrocelectomy was done (the fluid sucked and calcified tunica was excised) and the sample was sent for histopathology; histopathologic examination of the sample showed a cyst wall without cyst lining with fibrosis and extensive calcification (Figs. 4, 5, 6). Subsequently, the patient was commenced on ceftriaxone 1gm IV twice a day, scrotal elevation, wound care, and analgesics. The patient had unremarkable recovery and was discharged improved after 48 hours of hospital stay with no perioperative complications. On subsequent follow-up at the urology referral clinic, the wound healed well and the patient did not have any other complaints.

## Discussion

A hydrocele is a fluid collection within the tunica vaginalis of the scrotum or along the spermatic cord. In the adult population, filariasis, caused by *Wuchereria bancrofti*, accounts for most causes of hydrocele worldwide, affecting more than 100 million people, of whom 30% live in Africa [4]. Calcification of hydrocele sac/wall is a rare clinical entity. Kickham published “Calcified hydrocele of the tunica vaginalis testis: a case report” in 1935. In untreated, neglected, long-standing cases, the hydrocele may result in calcification of the tunica vaginalis. One study reported calcification of tunica vaginalis in a patient with long-standing hydrocele from a filariasis endemic geographic area and stated that recurrent attacks of epididymoorchitis resulting from filariasis might have led to hydrocele formation and subsequent calcification [5]. In another report by Koh *et al*., calcification of the tunica vaginalis in a patient with chronic hematocele was demonstrated by ultrasound (US) as a calcified shell in a 65-year-old male patient with history of trauma [6]. Kokotas *et al*. also reported a 72-year-old male patient in whom they ablated thick, hardened, and calcified tunica vaginalis. They stated that calcification was probably due to chronic irritation of the wall in the coexisting hydrocele [3]. An unnoticed minor trauma may have caused a hematocele, which may have led to testicular infarction and necrosis associated with calcification of tunica in some patients. The fibrosis may also be caused by a chronic irritant stimulus of the long-standing hydrocele [7].

Calcification of hydrocele sac is a rare presentation. The etiology of calcification of hydrocele sac is not clear, but most literature proposes that calcification is secondary to chronic irritation. *Schistosoma haematobium* is also responsible for tunica vaginalis calcification in endemic areas. Intrascrotal calcification in layers of the testis can also be seen in chronic diseases such as tuberculous epididymoorchitis, mostly after taking antitubercular treatment [8]. Scrotal calcification is a rare complication of childhood hydrocele testis, and it can occur secondary to a growing calcification reacting to the infarction due to the repeated torsion of the appendix epididymis [9].

The calcification had been shown on plain radiography of the pelvis as a calcified hydrocele sac. US is the first choice of investigation for any scrotal swelling to differentiate testicular lesions from extratesticular pathology. However, calcification of the wall may obscure the details of underlying pathology [1]. US is useful in infants and children in determining whether a scrotal mass is intratesticular (more likely malignant) or extratesticular. If extratesticular, the mass may be due to fluid or may contain fluid. The sonographic features of this fluid, including location, septation, and internal echoes as well as associated features such as adjacent Doppler flow, scrotal thickening, calcifications, and soft-tissue masses, can help to focus diagnostic considerations [10].

Grayscale ultrasonographic findings of tunica vaginalis calcification have been reported as a linear plaque with acoustic shadowing and dystrophic calcification of the tunica albuginea testis. Grayscale ultrasonography confirmed the dystrophic calcification of the tunica albuginea testis. In both tunica vaginalis and tunica albuginea testis calcification, no malignancy has been reported yet. Intrascrotal calcification can be seen in tuberculous epididymo-orchitis, especially during antituberculous chemotherapy [11, 12].

Treatment for primary hydrocele is open surgery, which is a common practice. Surgical excision of hydrocele sac is the treatment of choice in calcified hydrocele. This also differentiates other pathology of testes such as testicular malignancy, which is difficult to rule out clinically without radiological investigations, or histopathologic examination [8]. In our case, the diagnosis was made initially with the US scan and later confirmed with histopathology. The patient claimed that he was satisfied with the care he was provided.

## Conclusion

Calcification of the tunica vaginalis is very rare and is probably due to chronic irritation of the wall from the coexisting hydrocele. Diagnosis can be made using imaging studies or histopathology. Surgical excision of calcified hydrocele sac is the treatment of choice.

## Data Availability

The authors of this manuscript are willing to provide any additional information regarding the case report.

## Abbreviation

US: Ultrasound

## References

[1] Dave PK, Gupta V, Mishra R, Jain M, Bapat M, Tapadia R. Enlarged scrotum, an unusual cause-eggshell calcification of hydrocele on Lt with inguino-scrotal hernia and encysted hydrocele on Rt side, Imaging of a case & review.

[2] Goel A, Kumar P, Jain M, Singh G (2020). Eggshell calcification in a case of longstanding hydrocele. BMJ Case Rep.

[3] Kokotas N, Kontogeorgos L, Kyriakidis A (1983). Calcification of the tunica vaginalis. Br J Urol.

[4] Beyene AD, Kebede F, Mammo BM, Negash BK, Mihret A, Abetew S (2021). The implementation and impact of a pilot hydrocele surgery camp for LF-endemic communities in Ethiopia. PLoS Negl Trop Dis.

[5] Goel A, Singh V, Dalela D (2007). Calcification of tunica vaginalis in a case of longstanding hydrocele. Urology.

[6] Koh E, Kondoh N, Kiyohara H, Fujioka H, Kokado Y (1989). A case of chronic huge hematocele. Hinyokika Kiyo.

[7] Mutreja D, Murali M, Arya A. Pseudotumors of paratesticular region mimicking malignancy. Arch Int Surg. 2013; 13 (3):[about 3 p.].

[8] Barolia DK, Gupta SP, Sethi D, Sharma M. Eggshell calcification of hydrocele sac-A rare case.

[9] Gakiya M, Nakai H, Higuchi A, Shishido S, Kawamura T (1994). Scrotal calcification associated with hydrocele testis in a child: a case report. Hinyokika Kiyo.

[10] Chung SE, Frush DP, Fordham LA (1999). Sonographic appearances of extratesticular fluid and fluid-containing scrotal masses in infants and children: clues to diagnosis. AJR Am J Roentgenol.

[11] Conkbayır I, Yanik B, Keyik B, Hekimoglŭ B (2009). Eggshell calcification of the testis: ultrasonographic findings. J Ultrasound Med.

[12] Kickham CJE (1935). Calcified hydrocele of the tunica vaginalis testis: Case report. N Engl J Med.

